# Supplementary material for: Patterns of Intron Gain and Loss in Fungi
Source: PLoS Biol. 2004 Nov 30;2(12):e422. doi: 10.1371/journal.pbio.0020422 (PMC532390; doi:10.1371/journal.pbio.0020422)
Supplement: Table S1 — Also available at http://genes.mit.edu/NielsenEtAl/. (4.3 MB ZIP). [file pbio.0020422.st001.zip › NielsenEtAl/html/959.html]

AN1354.1.NCU01022.1.MG10652.1.FG10076.1


```
 CLUSTAL W (1.82) Multiple Sequence Alignments - Introns Inserted


Sequence 1: NCU01022.1	751 aa
Sequence 2: FG10076.1	719 aa
Sequence 3: MG10652.1	722 aa
Sequence 4: AN1354.1	744 aa
Alignment Length: 778 aa
Number Identitical Residues: 244 aa
Alignment Score (without introns) 14009


MG10652.1 	MGPRGRGGSRGGS--------------FRGGGS--------------RGRGRGRGRGGVN
NCU01022.1	MAIRGRGGDRGGGGRGGGGRGRGGAGGFRGRGGSRGGGSRGGARGASRGRGRGSSRGGAR
FG10076.1 	MPPRGRGGN------------------FRGRGG--------------RGRGRGGRGGKAT
AN1354.1  	MIVGQKEAEINEQ------------ALWILLSS--------------AVADAGSGVFSLT
          	*    : .. .              . :   ..                 . *       

MG10652.1 	-RFGK------R~PRFDSARVEDKEK2ES~EEESLSEAEEDSEVPES---ESSDESVAEE
NCU01022.1	GGFGPR-----S~SKFNDARLADKD-~EE2EDEEEDISEEESNVSEDEIEESEEEDEEED
FG10076.1 	SRFGP------N~RRFDGARLAENDG2LE~SSGSESESAPEDTVMED--LESEDEDDDEE
AN1354.1  	QIFRPAGLRRRS2IHYLTASLRTESS~SG~AEDQPEEQFEDGVQAEGLDDDVLSDSDESD
          	  *   .       ::  * :  :.      . . .    :.   *.   :  .:.  .:

MG10652.1 	APT-RPYMALLRSLSK-----GSGIKRRKLDHEPNNSEESGEDDSSESSADEDAPDDVCG
NCU01022.1	AQSGQPYMSLLKSFQS-----ATKTKKRKLDHPEEEGPNKVTKTKDDDNSDDEDNEKVTE
FG10076.1 	TASAKPYMALLQSFNNESN--APNSKRRKLDHKESAQSQPGEDSSSDEEGE-EGQDDPEK
AN1354.1  	QQSGQPYNELLQLLQAKTDSKGPARKKRKTSHQNKEGTEEAEN-VAEMTAVEEGEETLDA
          	  :.:**  **: :. .:.:...  *:** .*  .   :   .   :  . .:  :    

MG10652.1 	DVDLVDEPEE-DHDEIMEQERSDDEDEKDL~TDSFNSHFASPDEVEVAERIKSITNHEWV
NCU01022.1	DVDAVDEPEE-DPQDAPLEDLFDEDDDLDD~SDPFETHFTAPDEATFQARIKAIQANKWR
FG10076.1 	DIDRAEDEAE-DQVEEQVDDDDDSEDEENP~TDPFDVHFAHPDEDIVAKRVKSAQEGNWA
AN1354.1  	ELQQQEPDEEGDLEDGDVDAQEDSDDEESG1NEPFESHFSSPDESELSQKIKAASENKWK
          	:::  :   *.*  :   :   *.:*: .  .:.*: **: ***  .  ::*:    :* 

MG10652.1 	-SKRLPSKSARTVLTLPKQKDASDNASGPVPVTNPATLI~LKPKLKESITSQRPQLDASE
NCU01022.1	-TDRIAKNSNRIYYNTPETGDSAEQKL-PHSISGVGDLK~LKQRLAESMAKHT-EFDEAE
FG10076.1 	-TKRALIQTLRATVTYPAS--DAGSEV-PKPIAGLDGLQ~LKQKLKEISSRKIGEFSAVQ
AN1354.1  	NAKKELPGGLKIWRAIPDCG--QDDVPLLAAMKSMSSVK0VKRKLKSAVTERIPSLSGNV
          	.:.:      :     *       .     .: .   :  :* :* .  : :  .:.   

MG10652.1 	QALAAPLFQYYDTLYCQRTVANGHNLRRLVCLHALNHIFK2TRDRVIKNSAKLAKESADP
NCU01022.1	KAVAPLLFNYQDMLYCNRSVASSESIRRMACLHALNHVFK2TRDRVIKNNTKLQR---DD
FG10076.1 	RNLSPLIFNYNDVLFCDRTVRNSDSLRELTCLHALNHVFK2TRDRVIKNNYKLAKEGQDN
AN1354.1  	QQVSPYIFDYQDVLYGARTTSESADMRDMLAVHATNHVLK~TRDRVLKNNARVAKE-QDG
          	: ::. :*:* * *:  *:. .. .:* : .:** **::* *****:**. :: :.  * 

MG10652.1 	DLEFRDQGFTRPKVLMLLPTRNSCAKMIEMMCSLTETDQQENRKRFDDSYVTREKQKFSN
NCU01022.1	TLELRDQGFTRPKVLMILPTRQSCVKMVEMICKVAAPEQQENRKRFDDGYVDKST-KFSD
FG10076.1 	DLELRDQGFTRPKVLFLLPTRNSCLRMVNMIRDLCEPDQQENRKRFDDGYVDKEA-RFGA
AN1354.1  	DLELRDQGFTRPKVLYLLPTRQACVRVVESITRFFQPEQQENKKRFIDAFSASDD-PSWE
          	 **:*********** :****::* :::: :  .  .:****:*** *.:   .      

MG10652.1 	DKPTDFRDLFEGNADDMFRLGVKFTRKTIKFFSQFYNSDILLASPLGLRMAMGSTE~EKN
NCU01022.1	DKPEDFRDLFAGNDDDMFRLGMKFTRKTIKYFSQFYNSDIIFASPLGLRMAIGSEE~EKK
FG10076.1 	DRPADFKDLFEGSDDDMFRLGMKFTRKTVKYFSQFYNSDILFASPLGLRMAIGSEE~DKK
AN1354.1  	DKPQDFRELFGGNDDDMFRIGLKFTRKTLKFFTQFYTSDLILASPLGLRTIMDQAD2VKK
          	*:* **::** *. *****:*:******:*:*:***.**:::*******  :.. :  *:

MG10652.1 	-LDHDFLSSIEIVIMDQADAILMQNWEHVEHIFEHLNRQPKEAHGADFSRIRSWYLDGQS
NCU01022.1	KLDYDFLSSIELVIVDQADALLMQNWEHVEFIFEHLNLQPRDAHGCDFSRVRSWYLDDQA
FG10076.1 	-LDFDFLSSIEMVVVDQADALLMQNWEHVEFIFEHLNLQPKDAHGCDFSRVRNWYLEDWA
AN1354.1  	-RDHDFLSSVEVVIVDHTDALLMQNWDHVSYILKHLNLQPKEAHGCDFSRVRTWYLDNNA
          	  *.*****:*:*::*::**:*****:**..*::*** **::***.****:*.***:. :

MG10652.1 	KNYRQTVILSSFNNPDLSEVMRVHCHNWQGKIRIQPDYQGVIQQLG--VKVKQTFSRFDA
NCU01022.1	KYFRQTVIFSAFNTPELAELQRLYCHNWAGKARLQAEYPGVIQYLG--VKTRQTFSRFDA
FG10076.1 	KYFRQTIILSAFNTPELSELLRLHCHNWAGKVRLQPEYPGTLSQLG--VKAKQTFSRFQS
AN1354.1  	RYVRQLILLSSFITPEINSVFSTHMHNVFGKVKLNPIYNGAIAELPLPVPVKQTFTRFDC
          	:  ** :::*:* .*:: .:   : **  ** :::. * *.:  *  .* .:***:**:.

MG10652.1 	QTIIADPDARFEYFIKAVVPSLTKR------VKDSCGTLVFIPSYMDFVRVRNWFATSPA
NCU01022.1	ASIAADPDARFAYFTKAIVPTLVKRA-----AKDAAGTLIFIPSYLDFVRVRNYFANNPA
FG10076.1 	KSVDKDPDARFEYFTSAIIPSLAKR------AKDATGTLIFVPSYLDFVRVRNYFATSSS
AN1354.1  	LSPTKDPDARFKHFTTTILSTLVRNITTSRNRASAGGTLIFIPSYLDFVRLRNYFATSQQ
          	 :   ****** :* .:::.:*.:. ::: .  .: ***:*:***:****:**:**..  

MG10652.1 	AQSLSFGVISEHTEVSETSRARSHFVTGRQKVLLYTERCHHFWRHQLRGVRRVIFYGLPD
NCU01022.1	VESVTFGNISEYADTPEASRARSHFLTGRHRVLLYTERAHHFRRYAIKGVKRVIFYGLPD
FG10076.1 	VENVTFGAISEYTDVPEASRARSHFLSGRHRVLLYTERAHHFRRYQFRGVQRVIFYGLPD
AN1354.1  	TTNVSFGAISEYTEPREISRARSYFMTGRQSVLLYTERLHHFRRFRIRGVKRIIMYGMPD
          	. .::** ***:::  * *****:*::**: ******* *** *. ::**:*:*:**:**

MG10652.1 	NPVFYKEIAGGCLGRSEQDMTLEPGEGSVRAMFSKYDVMKLERVVGTPRVGKMLHEKG-D
NCU01022.1	NPIFYREIAGGYLQKSEQALMLEHGQGHVKVMFSKYDIMKLERIVGTKRAGKMITEQG-D
FG10076.1 	NPIFYTEIAGGYLSKSEQDLRLEPGQGTAKVVFSKYDVMKLERIVGSKRVGKMIQDRG-D
AN1354.1  	NPIFWGEIVG-FLG-LDPAGVVEAAEGGVRALFSKYDALKLERIVGTKRVGNMLREKGGD
          	**:*: **.*  *   :    :* .:* .:.:***** :****:**: *.*:*: ::*.*

MG10652.1 	TFDFV
NCU01022.1	TFDFV
FG10076.1 	TFEFI
AN1354.1  	TFTFM
          	** *:
```
